# Unveiling a Scaling and Universal Behavior for the Magnetocaloric Effect in Cubic Crystal Structures: A Monte Carlo Simulation

**DOI:** 10.1038/s41598-019-41321-y

**Published:** 2019-03-26

**Authors:** J. D. Alzate-Cardona, J. S. Salcedo-Gallo, D. F. Rodríguez-Patiño, C. D. Acosta-Medina, E. Restrepo-Parra

**Affiliations:** 1 Departamento de Física y Química, Universidad Nacional de Colombia, Manizales, Colombia; 2 Departamento de Matemáticas y Estadística, Universidad Nacional de Colombia, Manizales, Colombia

## Abstract

The magnetocaloric effect and the universal character for the magnetic entropy change regarding the cubic crystal structures (SC, BCC, FCC) were investigated, in a qualitative way, using Monte Carlo simulations. A classical Heisenberg Hamiltonian with nearest neighbors, and next nearest neighbors interactions was implemented. In order to compute the critical temperature of the system depending on the coordination number, it was calculated the dependence of the magnetization and magnetic susceptibility as a function of temperature. Magnetic field dependence on the magnetization for isothermal processes was performed considering a magnetocrystalline anisotropy term. In this way, the magnetic entropy change (Δ*S*_*m*_) was computed. Results show that the rescaled Δ*S*_*m*_ as well as the exponent (*n*) characterizing the field dependence of the magnetic entropy change curves, collapse onto a single curve for the studied crystal structures. By this reason, it can be assured that Δ*S*_*m*_ exhibits a universal behavior regarding the strength and contribution of the magnetic exchange energy to the total magnetic energy.

## Introduction

The magnetocaloric effect (MCE) consists in the thermal response of a material when subjected to a magnetic field change and it is an intrinsic property of all magnetic materials. MCE in magnetic systems with second- or first- order phase transitions, near room temperature, has attracted significant interest in recent years due to their potential applications on magnetic refrigeration^1,2^. Magnetic refrigeration techniques have a larger efficiency and a smaller footprint on the environment, compared to conventional compression/expansion of gas techniques. Therefore, it is important to properly describe, both theoretically and experimentally, the field and temperature dependence of the magnetic entropy change (Δ*S*_*m*_) in magnetic materials. Materials suitable for magnetic refrigeration devices should operate at low, or moderate magnetic fields, in a range (1 − 2*T*)^3^. Perovskites are materials of great interest since they exhibit a large MCE. This type of materials crystallize into a distorted form of a body-centered cubic (BCC) crystal structure^4,5^. Likewise, there are other materials which crystallize into a cubic type structure, and that also exhibit MCE^6–8^. Both, experimental and computational studies, have been performed in order to comprehend the MCE in these materials^9–13^.

In recent years, it has been invested a great effort on correlating Δ*S*_*m*_ with the critical exponents of the ferromagnetic-paramagnetic phase transition^14–17^; then, by knowing the denominated phenomenological universal curve for Δ*S*_*m*_, it is possible to predict the behavior of Δ*S*_*m*_ for ferromagnetic alloys of the same compositional series or for the same material at different applied magnetic fields.

There are several cases where standard techniques like the Kouvel-Fisher method do not work properly^18,19^. Therefore, scaling laws for Δ*S*_*m*_ have been proposed for determining the critical exponents of a material^2,14–18,20^. The theoretical background of the MCE universal behavior relies on the scaling of second-order phase transitions. However, from a theoretical point of view, obtaining the universal curve requires the knowledge of the critical exponents of the material and its equation of state, which is a situation rather unlikely, especially when first undergoing with a new material in the laboratory^16^. Franco *et al*.^20^ proposed a phenomenological procedure to describe the universal behavior of Δ*S*_*m*_ in compounds with second-order phase transition, which can be followed without this *a priori* knowledge. In this way, it is possible to employ the universal curve in the characterization of new materials, such as a screening procedure of the performance of materials, as well as a method for making extrapolations to temperatures or fields not available in the simulated range or in the laboratory.

For materials of the same compositional series, it is likely to obtain different crystal structures and different exchange energy constants by slightly changing its composition. Then, the contribution of this work is focused on the study, by means of Monte Carlo simulations, of the universal behavior and scaling laws of Δ*S*_*m*_ for different cubic crystal structures with different coordination number, and to relate them by means of the exponent of the field dependence of Δ*S*_*m*_.

This work is presented as follows: In section II, we describe the model and method, the thermodynamics of the magnetocaloric effect as well as the formulas to compute the physical quantities of interest. In section III, we present the numerical results and discussion and finally, in section IV, we present the conclusions.

## Model and Method

Three cubic structures were studied: simple cubic (SC), body-centered cubic (BCC), and face-centered cubic (FCC) lattices. Periodic boundary conditions were imposed to the system. The size of each lattice was chosen so that all the simulated structures had approximately the same amount of ions, because the magnetocaloric properties depend on the size and the amount of ions within the structure^21^. The total number of ions was set at *N* ≈ 4000, which corresponded to a system size of *L* × *L* × *L*, where *L* was fixed at 16, 13, and 10 unit cells (uc), being *N* = 4096, 4394, and 4000 ions, for SC, BCC, and FCC lattices, respectively.

The magnetic behavior of the system was simulated using a classical Heisenberg Hamiltonian, which included terms for the exchange interaction of the magnetic moments with their nearest neighbors (NN) and their next nearest neighbors (NNN), magnetocrystaline anisotropy, and the interaction of the magnetic moments with an external magnetic field. It is worth to mention that, regarding the crystal structure, the coordination number (*z*) takes the values of 6, 8, and 12 NN for the SC, BCC, and FCC lattices, respectively. Regarding the NNN interactions, each structure has a total number of 12, 6, and 6 NNN for the SC, BCC, and FCC lattices, respectively. The Hamiltonian can be expressed as

1 $$ {\mathcal H} =-\,{J}_{{\rm{NN}}}\,\sum _{\langle i,j\rangle }\,{{\boldsymbol{S}}}_{i}\cdot {{\boldsymbol{S}}}_{j}-{J}_{{\rm{NNN}}}\sum _{\{i,j\}}\,{{\boldsymbol{S}}}_{i}\cdot {{\boldsymbol{S}}}_{j}-{k}_{{\rm{an}}}\,\sum _{i}\,{({{\boldsymbol{S}}}_{i}\cdot {\hat{{\boldsymbol{n}}}}_{i})}^{2}-H\sum _{i}\,{{\boldsymbol{S}}}_{i}\cdot \hat{{\boldsymbol{k}}}$$

where ***S***_*i*_ and ***S***_*j*_ are the directions of the magnetic moments in the sites *i* and *j*, respectively, with |***S***_*i*_| = |***S***_*j*_| = 1. Note that the first sum runs over the NN, and the second one runs over the NNN, where *J*_NN_ and *J*_NNN_ are the respetive exchange constants, with *J*_NN_ = 1.0. *H* is the magnetic field intensity, $\hat{{\boldsymbol{k}}}$ is the unitary vector in the *z*-direction, *k*_an_ is the anisotropy constant, and ${\hat{{\boldsymbol{n}}}}_{i}$ is the anisotropy vector, which was chosen in a perpendicular direction with respect to the applied magnetic field, *x*-direction. The simulations were carried out using V_EGAS_^22^, which is an open source software package for the atomistic simulations of magnetic materials employing the Monte Carlo method based on the Metropolis algorithm. The simulations were accomplished using 1 × 10^4^ Monte Carlo steps (MCS), rejecting the first 0.5 × 10^4^ MCS for relaxation. In order to compute error bars, the simulations were performed using 5 different initializations.

The magnetic susceptibility (*χ*) was calculated as

2 $$\chi =\frac{\langle {M}^{2}\rangle -{\langle M\rangle }^{2}}{{k}_{B}T}$$

where *k*_*B*_ is the Boltzmann constant and *T* is the absolute temperature. The critical temperature (*T*_*c*_) was calculated as the temperature at which the magnetic susceptibility exhibits a maximum value.

The total magnetization was calculated as

3 $${\boldsymbol{M}}=\frac{1}{N}\sum _{i}^{N}\,{{\boldsymbol{S}}}_{i}$$

and the total normalized magnetization was evaluated as

4 $$M={({\boldsymbol{M}}\cdot {\boldsymbol{M}})}^{\frac{1}{2}}$$

The change in the magnetic entropy, using the Maxwell relations, can be expressed as^23^

5 $${\rm{\Delta }}{S}_{m}={\int }_{0}^{{H}_{f}}{(\frac{\partial M}{\partial T})}_{H}dH$$

where *H*_*f*_ is the superior limit of the applied magnetic field intensity. In the case of a discrete field, Δ*S*_*m*_ can be approximated by^24^

6 $${\rm{\Delta }}{S}_{m}(\frac{{T}_{1}+{T}_{2}}{2})\approx (\frac{1}{{T}_{2}-{T}_{1}})[{\int }_{0}^{{H}_{f}}\,M({T}_{2},H)dH-{\int }_{0}^{{H}_{f}}\,M({T}_{1},H)dH]$$

where *T*_2_ > *T*_1_. In order to compute Δ*S*_*m*_ employing the Eq. 6, it is necessary to calculate the magnetization of the material as a function of the applied magnetic field at small discrete steps, for several isothermal processes.

The aforementioned method proposed by Franco *et al*.^20^ consists in normalizing the Δ*S*_*m*_ curves with respect to their maximum and rescaling the temperature axis in a different way, by means of a variable *θ*, below and above *T*_*c*_, by imposing that the position of two reference points in the curve corresponds to *θ* = ±1, as follows

7 $$\theta =(\begin{array}{cc}{}^{-(T-{T}_{c})}/({T}_{{r}_{1}}-{T}_{c}) & T\le {T}_{c}\\ {}^{(T-{T}_{c})}/({T}_{{r}_{2}}-{T}_{c}) & T > {T}_{c}\end{array}$$

where ${T}_{{r}_{1}}$ and ${T}_{{r}_{2}}$ are the temperatures of the two reference points. For this study, they have been selected as those corresponding to $0.5|{\rm{\Delta }}{S^{\prime} }_{m}|$, being ${\rm{\Delta }}{S^{\prime} }_{m}={}^{{\rm{\Delta }}{S}_{m}}/{\rm{\Delta }}{S}_{m}^{{\rm{pk}}}$, where ${\rm{\Delta }}{S}_{m}^{{\rm{pk}}}$ is the value of the maximum of the |Δ*S*_*m*_| curve.

It can be assumed that the field dependence of Δ*S*_*m*_ follows the power law with the field as^20^

8 $$|{\rm{\Delta }}{S}_{m}|\propto {H}^{n}$$

being *n* a parameter which depends on temperature and can be locally calculated as

9 $$n=\frac{d\,\mathrm{ln}\,|{\rm{\Delta }}{S}_{m}|}{d\,\mathrm{ln}\,H}$$

At low temperatures, well below *T*_*c*_, *n* should have a value that tends to 1, which indicates that although the magnetization curves depend on temperature, at these temperatures, this dependence is essentially field independent. At temperatures well above *T*_*c*_, *n* should tend to 2 as a consequence of the Curie-Weiss law. At *T* = *T*_*c*_, *n* shows a global minimum, corresponding to the value of *n*(*T*_*c*_). Oesterreicher and Parker predicted that *n*(*T*_*c*_) = 2/3 for the mean field case^16,25^. However, for other cases such as Heisenberg model, the value of *n*(*T*_*c*_) is different from the one deduced for the mean field and it is closely related to the critical exponents of the model and material^20^. It was found that the value of *n*(*T*_*c*_) is equal to

10 $$n({T}_{c})=1+\frac{\beta -1}{\beta +\gamma }$$

where *β* and *γ* are critical exponents, related with the magnetization and susceptibility, which depend on the used model and material.

## Results and Discussion

**Figure 1:**
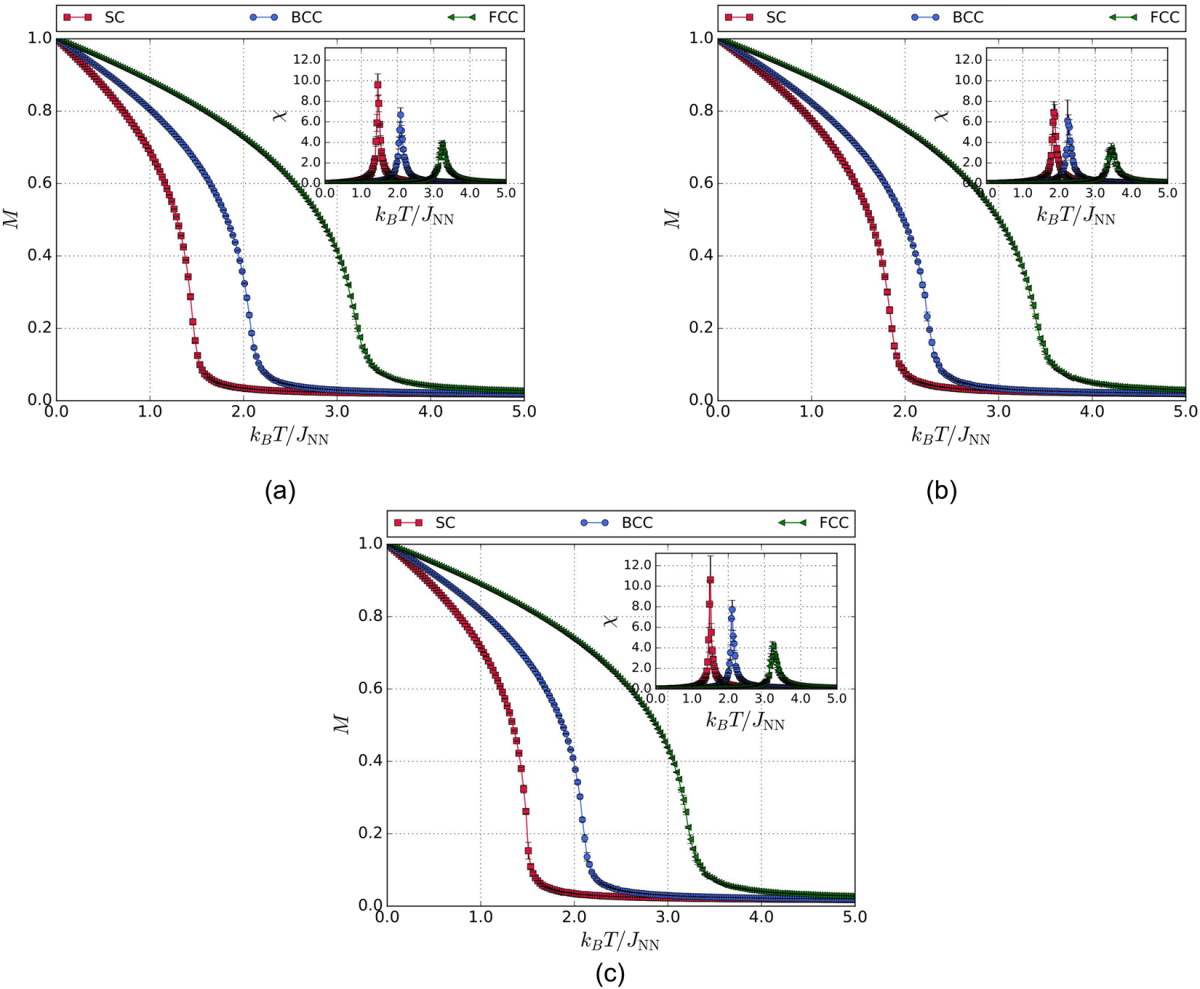
Temperature dependence of the magnetization and magnetic susceptibility for SC, BCC, and FCC lattices for (**a**) ${}^{{J}_{{\rm{NNN}}}}/{J}_{{\rm{NN}}}=0.0$, and ${}^{{k}_{{\rm{an}}}}/{J}_{{\rm{NN}}}=0.0$. (**b**) ${}^{{J}_{{\rm{NNN}}}}/{J}_{{\rm{NN}}}=0.1$, and ${}^{{k}_{{\rm{an}}}}/{J}_{{\rm{NN}}}=0.0$. (**c**) ${}^{{J}_{{\rm{NNN}}}}/{J}_{{\rm{NN}}}=0.0$, and ${}^{{k}_{{\rm{an}}}}/{J}_{{\rm{NN}}}=0.1$.

Figure 1 shows the temperature dependence of the magnetization and magnetic susceptibility curves for SC, BCC, and FCC crystal lattices taking into account the interactions of NN without anisotropy, NNN, and NN with anisotropy, respectively. It can be observed that, as the coordination number increases, the critical temperature increases. This is due to the fact that the exchange energy increases as the coordination number increases and, thus, more thermal energy is required to break the magnetic ordering imposed by the exchange interactions and to reach the paramagnetic regime. It can also be seen that the peak of the susceptibility curves decreases when the interaction of the NNN (Fig. 1b) is taken into account. This behavior could be explained from the fact that the role of the NNN interactions is to increase the magnetic ordering in the system by increasing the exchange energy contribution to the total magnetic energy, thus, reducing the thermal fluctuations of the magnetization. As a consequence, even more thermal energy is required to break the magnetic ordering imposed by the exchange interactions. Moreover, in Fig. 1c, it can be observed that the peak height of the magnetic susceptibility is greater when considering the magnetocrystalline anistropy term in the hamiltonian. Also, the error bars are larger, meaning that thermal fluctuations gain significance since there is a competition between both exchange and anisotropic energies.

**Figure 2:**
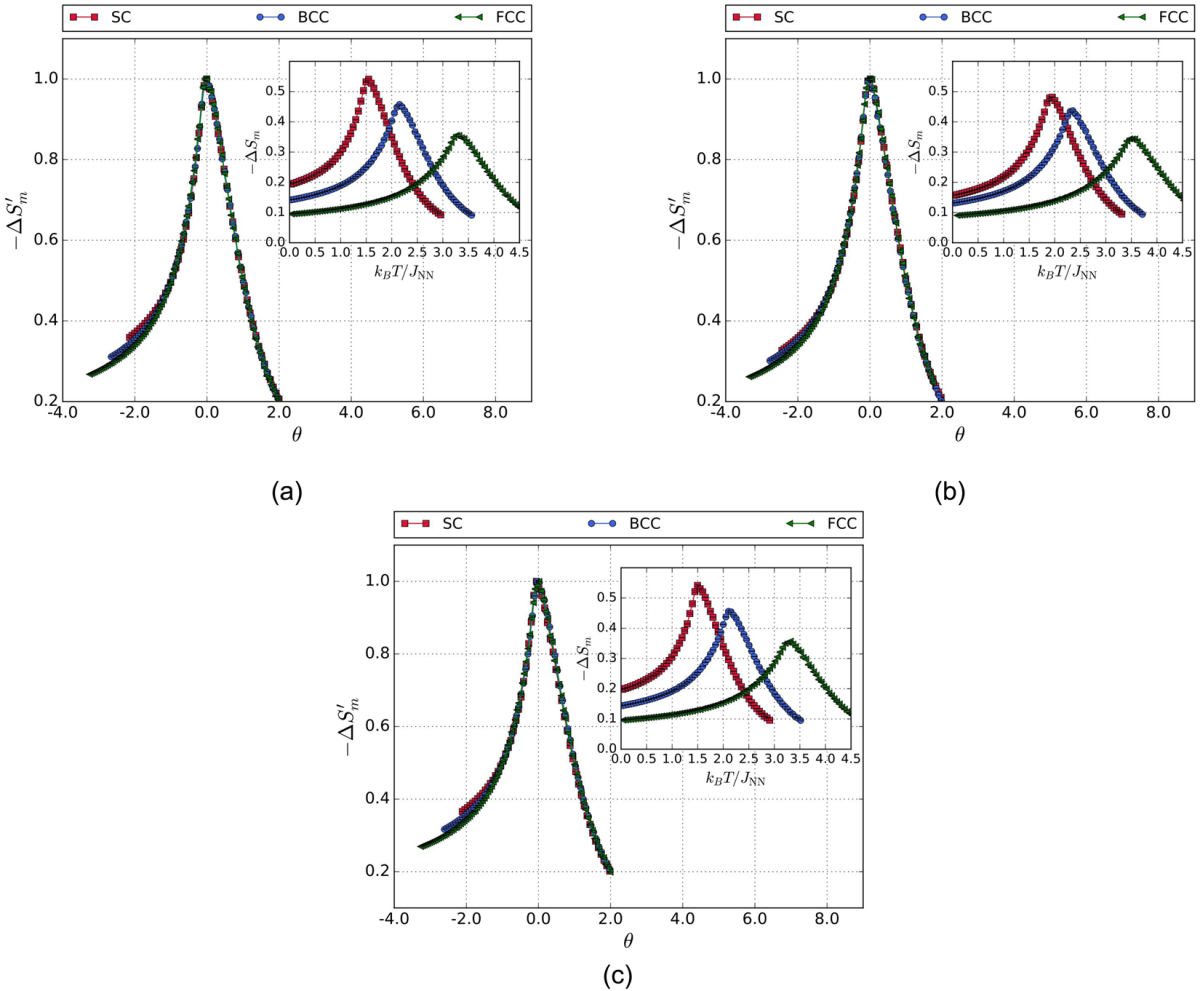
Insets: temperature dependence of the magnetic entropy change curves for SC, BCC, and FCC lattices. Main figures: master curve behavior for the magnetic entropy change for (**a**) ${}^{{H}_{f}}/{J}_{{\rm{NN}}}=1.0$, ${}^{{J}_{{\rm{NNN}}}}/{J}_{{\rm{NN}}}=0.0$, and ${}^{{k}_{{\rm{an}}}}/{J}_{{\rm{NN}}}=0.0$. (**b**) ${}^{{H}_{f}}/{J}_{{\rm{NN}}}=1.0$, ${}^{{J}_{{\rm{NNN}}}}/{J}_{{\rm{NN}}}=0.1$, and ${}^{{k}_{{\rm{an}}}}/{J}_{{\rm{NN}}}=0.0$. (**c**) ${}^{{H}_{f}}/{J}_{{\rm{NN}}}=1.0$, ${}^{{J}_{{\rm{NNN}}}}/{J}_{{\rm{NN}}}=0.0$, and ${}^{{k}_{{\rm{an}}}}/{J}_{{\rm{NN}}}=0.1$.

Isothermal simulations of the magnetization as a function of the applied magnetic field were performed in order to compute Δ*S*_*m*_. The insets of Fig. 2a,b show Δ*S*_*m*_ as a function of the temperature for SC, BCC, and FCC structures, taking into account the interactions of the ions with their NN without anisotropy, NNN, and NN with anisotropy, respectively. It can be noted that the values of ${\rm{\Delta }}{S}_{m}^{{\rm{pk}}}$ were found to be around *T*_*c*_, as it is expected. This is because near *T*_*c*_, the magnetic moments pass from having a magnetic structure, governed by the exchange interactions between ions, to be in a paramagnetic regime, which is a disordered magnetic configuration. As a result, −Δ*S*_*m*_ has a maximum due to the abrupt change in the magnetization of the material. On the other hand, the value of ${\rm{\Delta }}{S}_{m}^{{\rm{pk}}}$ decreases as the coordination number increases. Also, the values of ${\rm{\Delta }}{S}_{m}^{{\rm{pk}}}$ are smaller when considering NNN interactions, when compared with NN interactions. This is because, since *H*_*f*_ was fixed at the same value for the −Δ*S*_*m*_ curves $({}^{{H}_{f}}/{J}_{{\rm{NN}}}=1.0)$, the influence of the magnetic field in Δ*S*_*m*_ will be more significant as the structure has lower coordination number, or if the interactions with the next nearest neighbors are not considered. As a result, the contribution of the exchange energy will be lower within the structure. Moreover, below *T*_*c*_, −Δ*S*_*m*_ increases monotonically and, for values above *T*_*c*_, −Δ*S*_*m*_ decreases monotonically, which is in agreement with the standard behavior of magnetic refrigerants.

The main plot of Fig. 2a–c show the universal behavior of the magnetocaloric effect for different cubic crystal structures, considering the interactions of NN without anisotropy, NNN, and NN with anisotropy, respectively. It is possible to observe that, for every figure, all the rescaled curves converge onto the same curve. This means that the second-order phase transition in these studied crystal structures are driven by the same phenomenological behavior. In this way, there is not only a universality and scaling laws for Δ*S*_*m*_ regarding the magnetic field, as proposed by Franco *et al*.^3,15–18,20,26^, but there is also a universality and scaling laws for materials regarding the contribution and the strength of the magnetic exchange energy to the total magnetic energy, as long as these ferromagnetic alloys exhibit a second-order phase transition. Furthermore, it can be seen that the master curve of Δ*S*_*m*_ has a few discrepancies at low temperatures. This can be explained from the fact that, at low temperatures, the magnetic behavior of the system is mainly driven by the exchange interaction.

**Figure 3:**
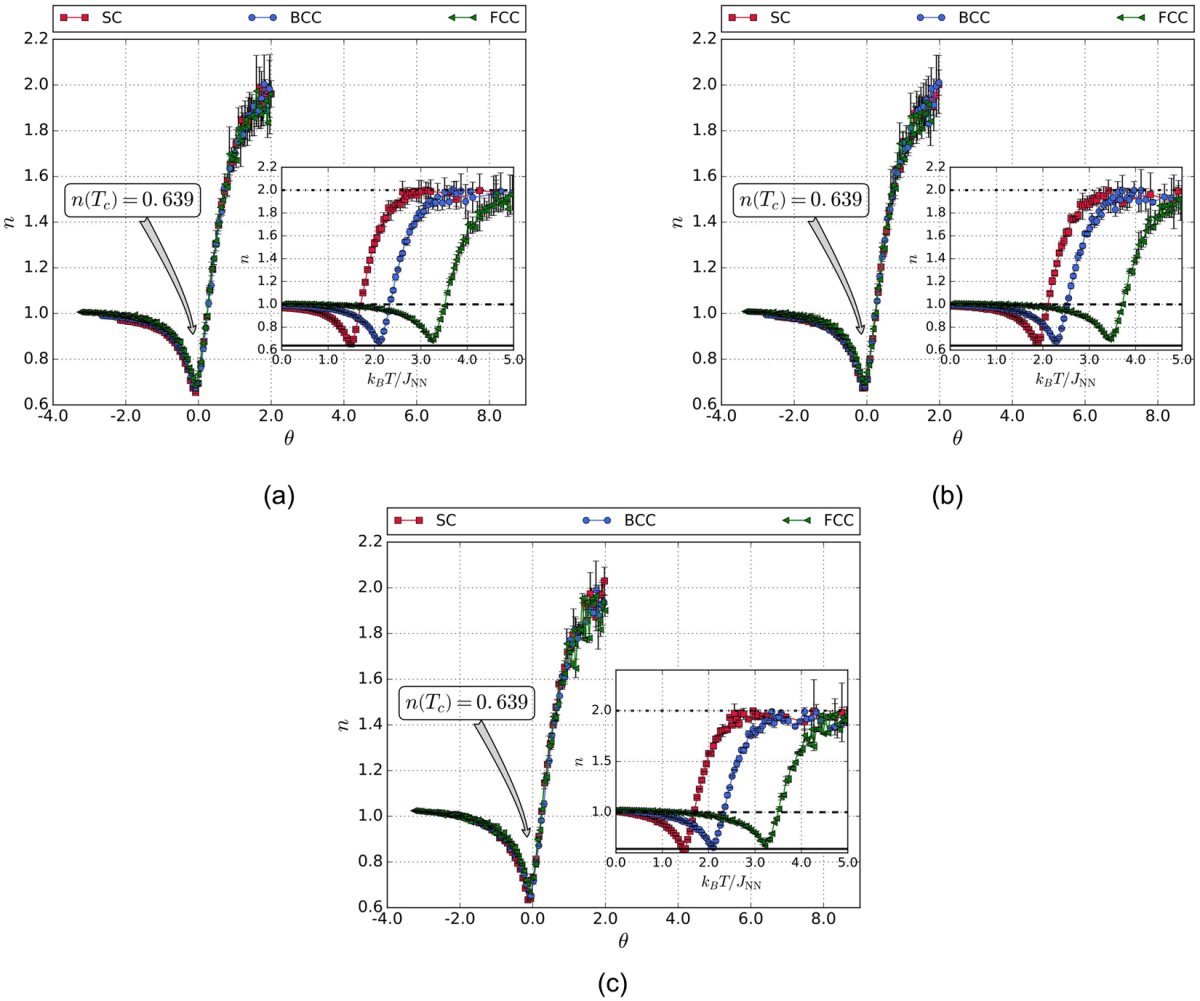
Insets: temperature dependence of the exponent characterizing the field dependence of SC, BCC, and FCC lattices. Main figures: master curve behavior for the exponent n for (**a**) ${}^{{H}_{f}}/{J}_{{\rm{NN}}}=1.0$, ${}^{{J}_{{\rm{NNN}}}}/{J}_{{\rm{NN}}}=0.0$, and ${}^{{k}_{{\rm{an}}}}/{J}_{{\rm{NN}}}=0.0$. (**b**) ${}^{{H}_{f}}/{J}_{{\rm{NN}}}=1.0$, ${}^{{J}_{{\rm{NNN}}}}/{J}_{{\rm{NN}}}=0.1$, and ${}^{{k}_{{\rm{an}}}}/{J}_{{\rm{NN}}}=0.0$. (**c**) ${}^{{H}_{f}}/{J}_{{\rm{NN}}}=1.0$, ${}^{{J}_{{\rm{NNN}}}}/{J}_{{\rm{NN}}}=0.0$, and ${}^{{k}_{{\rm{an}}}}/{J}_{{\rm{NN}}}=0.1$.

Figure 3a–c show the scaling and universal behavior for the exponent characterizing the field dependence of Δ*S*_*m*_, considering the interactions of the ions with their NN without anisotropy NNN, and NN with anisotropy, respectively. It can be seen that, at temperatures well below *T*_*c*_, *n* → 1. This indicates that, although the magnetization curves depend on temperature at these temperatures, this dependence is essentially field independent. Furthermore, for values well above *T*_*c*_, *n* → 2. This indicates that the change in the magnetic entropy has a quadratic field dependence, as a consequence of the well known Curie-Weiss law^27^. For *T* = *T*_*c*_, *n* → 0.639 (solid horizontal line in Fig. 3). This result is in agreement with the critical behavior reported, using the values of the critical exponents *β* and *γ* for the Heisenberg model. The aforementioned values were found to be *β* = 0.367 and *γ* = 1.388^26^. Therefore, using Eq. 10, *n*(*T*_*c*_) = 0.639. It can also be seen from Fig. 3b that the scaling and universal behavior of *n* holds up even when considering the interaction of the ions with their next nearest neighbors.

Figures 2c and 3c show the universality and scaling behavior for the temperature dependence of Δ*S*_*m*_ and *n*, respectively. The simulations were carried out rejecting the interactions of the magnetic moments with their NNN (*J*_NN_/*J*_NN_ = 0.0), and considering the influence of the magnetocrystalline anisotropy term $({}^{{k}_{{\rm{an}}}}/{J}_{{\rm{NN}}}=0.1)$. The uniaxial anisotropy axis was chosen in the *x*-direction, perpendicular to the applied magnetic field, with the aim of avoid blocking of the magnetic moments at low temperatures. The mentioned figures show that both temperature dependence of Δ*S*_*m*_ and *n* keep the universality and scaling behavior. Therefore, as the anisotropic term is weak in energy, for this systems, this term does not play an important role, and does not affect the universality nor the scaling behavior studied in this work.

## Conclusions

The universal behavior of the magnetocaloric effect regarding the crystal structure, related with the strength and contribution of the magnetic exchange energy, was investigated by means of Monte Carlo simulations using a Heisenberg model with nearest neighbors and next nearest neighbors interactions. Simulations of the magnetization as a function of the applied magnetic field were carried out at isothermal processes to find the temperature dependence of −Δ*S*_*m*_ for different values of the magnetic applied field, for SC, BCC, and FCC structures. It was found that the value of $|{\rm{\Delta }}{S}_{m}^{{\rm{pk}}}|$ decreases as the coordination number increases. It was showed that the rescaled −Δ*S*_*m*_ curves for different cubic crystal structures follow the same behavior, meaning that there exists a scaling law for −Δ*S*_*m*_ for the crystal structures investigated in this work. Finally, the temperature dependence of *n* was obtained for each structure and different anisotropy constants, moreover, the rescaled *n*(*T*) curves lies in the same curve, and the exponent *n*(*T*_*c*_) is in agreement with the critical exponents for the used model,confirming the universality and scaling behavior of −Δ*S*_*m*_ for the studied magnetic systems.

## Data Availability

The datasets generated during the current study are available from the corresponding author on reasonable request.
